# Editorial: Synthetic data for computer vision in agriculture

**DOI:** 10.3389/fpls.2023.1277073

**Published:** 2023-09-18

**Authors:** Manya Afonso, Valerio Giufrida

**Affiliations:** ^1^ Biometris, Wageningen Plant Research, Wageningen University and Research, Wageningen, Netherlands; ^2^ School of Computing, Edinburgh Napier University, Edinburgh, United Kingdom

**Keywords:** synthetic data, computer vision in agriculture, segmentation, deep learning, object detection

Availability of good quality data is often the deciding factor in the application of deep learning to computer vision problems in agriculture, as in other domains. Synthetic data, if realistic enough, can offer a solution in this case. Due to the inherent nature of creating or simulating data, the respective ground truth, whether image level labels, bounding boxes, or instance masks, can be prepared automatically. In fact, synthesis can be thought of as the opposite of segmentation, and therefore lends itself to generative models, such as generative adversarial networks (GANs) Goodfellow et al. (2020). It has been previously shown that synthetically generated images of sweet pepper plants using 3D modeling for the plant structure and with GANs for improving the realsim of the projected 2D images Barth et al (2018a; 2018b). when used as the initial training set for sweet pepper detection models, led to an improvement in the overall detection performance across different datasets Afonso (2019). This suggests that training on synthetic data can make the models learn some latent characteristics, which can then be refined by training on the respective empirical data.

The aim of this Research Topic is to showcase some of the recent applications of the use of synthetic data in agricultural computer vision problems. Here, we highlight some of the topics from the following contributions.

In Wang et al., a dataset for training a YOLO Redmon et al. (2016) based object detector for pear flowers was developed synthesizing extracted flowers on the backgrounds of the target use case. The modified model called YOLO-PEFL differs from YOLOv4 in that the backbone architecture used is ShuffleNetv2 Ma et al. (2018) combined with the SENet model, and obtained an Average Precision of 96.71%, with all metrics better than those obtained by training only on natural data.

Image enhancement transforms were used in Yu et al. to generate a training dataset for the detection of brown leaf spot, frog-eye leaf spot, and phyllosticta leaf spot in soybeans. A modified resnet18 He et al. (2016) model including an attention layer was developed, which when trained on this dataset resulted in an average disease recognition accuracy of 98.49%.

Another work on leaf disease detection Sapoukhina et al. generated synthetic fluorescent images of arabidopsis plants by statistical quantification of pixel values corresponding to healthy and diseased leaves, and using the parameters obtained to generate appropriate pixel values given an input mask indicating the healthy regions and leaf lesions. A U-Net model for segmenting the lesions trained on this dataset showed 0.793 recall and 0.723 average precision on an empirical fluorescent test dataset.

Two works dealt with the problem of segmentation of roots. In Li et al., a model for 3D semantic segmentation of roots was proposed, based on U-Net with modified layers to reduce the training volume, to improve the feature utilization capability and to improve the segmentation accuracy of the object through

multi-scale features and attention fusion. In addition, the dice-focal loss function was modified to be able to avoid data imbalance problems related to the backgrounds such as between the root system and soil. Experimental results on a test set of the peanut root images obtained a root segmentation accuracy of 0.9917, an Intersection Over Union of 0.9548, and an F1-score of 95.10. Transfer Learning for segmentation of corn roots was also reported.

A method for 3D segmentation of cassava roots Alle et al. utilized a custom CNN architecture with a spatial pyramid pooling layer to deal with the variance in the root structures, along with a dataset obtained by recursively refining ground truth segmentation obtained using the root force algorithm Gerth et al. (2021) in the weakly supervised framework Khoreva et al. (2017). Experimental results showed that finer root structures could be segmented using this approach coupled with locally adapting the field-of-view.

## Final comments

The articles presented in this Research Topic show different approaches to generating synthetic data, such as relatively simple pixel colorspace transformations, overlaying objects of interest onto target backgrounds, statistical modeling of pixel values, and recursively refining baseline segmentations. The deep learning problems included segmentation and object detection, with disease detection also formulated as a segmentation problem for the diseased/damaged regions. The reported results show that improvements in the metrics of interest could be obtained using models trained on the synthetic data, thus making a strong case for using synthetic data as a starting point for the introduction of deep learning in these kind of computer vision problems in situations hitherto without annotated data.

## Author contributions

MA: Writing – original draft, Writing – review & editing. VG: Writing – review & editing.
